# Light-Responsive Micelles Loaded With Doxorubicin for Osteosarcoma Suppression

**DOI:** 10.3389/fphar.2021.679610

**Published:** 2021-06-18

**Authors:** Jiayi Chen, Chenhong Qian, Peng Ren, Han Yu, Xiangjia Kong, Chenglong Huang, Huanhuan Luo, Gang Chen

**Affiliations:** ^1^ Bengbu Medical College, Bengbu, China; ^2^ Jiaxing Key Laboratory of Basic Research and Clinical Translation on Orthopedic Biomaterials, Department of Orthopaedics, The Second Affiliated Hospital of Jiaxing University, Jiaxing, China

**Keywords:** light-responsive nanoparticles, micelles, doxorubicin, targeted therapy, osteosarcoma

## Abstract

The enhancement of tumor targeting and cellular uptake of drugs are significant factors in maximizing anticancer therapy and minimizing the side effects of chemotherapeutic drugs. A key challenge remains to explore stimulus-responsive polymeric nanoparticles to achieve efficient drug delivery. In this study, doxorubicin conjugated polymer (Poly-Dox) with light-responsiveness was synthesized, which can self-assemble to form polymeric micelles (Poly-Dox-M) in water. As an inert structure, the polyethylene glycol (PEG) can shield the adsorption of protein and avoid becoming a protein crown in the blood circulation, improving the tumor targeting of drugs and reducing the cardiotoxicity of doxorubicin (Dox). Besides, after ultraviolet irradiation, the amide bond connecting Dox with PEG can be broken, which induced the responsive detachment of PEG and enhanced cellular uptake of Dox. Notably, the results of immunohistochemistry *in vivo* showed that Poly-Dox-M had no significant damage to normal organs. Meanwhile, they showed efficient tumor-suppressive effects. This nano-delivery system with the light-responsive feature might hold great promises for the targeted therapy for osteosarcoma.

## Introduction

Cancer is a disease that seriously affects human health, being one of the primary causes of death all over the worldwide (Tang et al., 2011; Gohlke et al., 2016; Jiang et al., 2020). Osteosarcoma is a highly aggressive tumor originating from the long bones, which is the most frequent primary malignant tumor in children and adolescents, with a wide spectrum of morphology (Hogendoorn et al., 2010; Yen et al., 2018; Sadykova et al., 2020). The annual morbidity rate in Europe is 2–30 per million people, and there are approximately 400–1,000 newly diagnosed cases of osteosarcoma in the United States each year (Mirabello et al., 2009; Kaatsch, 2010; Casali et al., 2018). It has a strong propensity to metastasize to the lungs, and localized limb osteosarcoma treated with surgery alone has a poor prognosis (< 20% 2-years survival) (Whelan et al., 2012; Morrow et al., 2018). Presently, the standard clinical treatment for osteosarcoma includes surgery and systemic chemotherapy (Kawano et al., 2010; Turner et al., 2014; Freyer et al., 2017). Oral drugs or intravenous, with a combination of various chemotherapy drugs, are administered to patients both preoperatively and postoperatively (Anninga et al., 2011). Although this type of surgery combined with chemotherapy significantly improves the 5-years survival rate for osteosarcoma patients to 70%, the overall therapeutic effect remains unsatisfactory with a high mortality rate (Chen et al., 2019; Gerakis et al., 2019; Gomez-Brouchet et al., 2019).

The anthracycline doxorubicin (Dox) has been the cornerstone of anticancer therapy over the past 50 years (Andreadis et al., 2007; Tarpey et al., 2019; Shen and Cheng, 2020). However, although Dox is widely used in the treatment of various cancers with promising efficacy, it is highly toxic and can cause dose-dependent irreversible serious cardiotoxicity and liver damage with long-term use (Hartmann et al., 2020; Mitry et al., 2020; Prasanna et al., 2020; Qiao et al., 2020). As a result, the clinical use of many of such chemotherapeutic agents is limited (Takagi et al., 2014; Li et al., 2015; Shi et al., 2020). Also, the intravenous administration of traditional chemotherapy will not only produce significant biotoxic side effects on the relevant metabolic systems of the human body, but also lead to the ineffective distribution of chemotherapeutic drugs, which is also an important reason for the waste of medical resources and inefficiency of chemotherapy(Zhang et al., 1997; Fan et al., 2005; Kumar et al., 2015). It is also reported that more than 30% of patients with osteosarcoma are either resistant to present chemotherapy regimens or develop critical life-threatening complications, and eventually succumb to metastases and death (Berrino et al., 2007). Therefore, looking for a new preparation that can improve the targeting of drugs to osteosarcoma, improve bioavailability, reduce side effects has important practical significance for the treatment of patients with osteosarcoma (Ghaffarian et al., 2016).

In recent years, with the rapid development of nanomedicine, a variety of nano-drug-loaded sustained-release particles have been intensively studied in the field of biomedical application (Suna et al., 2020; Hu et al., 2021; Lakshmanan et al., 2021). The nano-drug delivery system has a unique core-shell structure, and as a hydrophobic drug carrier, it has the characteristics of good biocompatibility, high drug loading rate, high circulation time *in vivo*, and so on (Cheng et al., 2016; Yousefpour and Yari, 2017). The nano-carrier can achieve the controllable sustained release of various chemotherapeutic drugs in the drug delivery environment according to the design needs so that the active molecules form a highly dominant concentration in the local tumor and effectively kill tumor cells (Hanafy et al., 2018). Moreover, it substantially avoids the whole-body distribution of biotoxic molecules and effectively avoids all kinds of toxic and side effects of traditional chemotherapy drug delivery, so it is the most appropriate dosage form for carrying anticancer drugs (De Vos et al., 2014; Pradeep et al., 2017; Makeen et al., 2020). Nano-micelles have achieved great success in the current cell and animal experiments, but due to the defects that the drug is released ahead of time and the drug carrier cannot fully release the drug when it reaches the focus, the clinical application of drug-loaded micelles is limited (Sethuraman et al., 2021). To this end, the researchers designed and synthesized stimulus-responsive micelles to achieve controlled release of the drug at the focal site. Common stimulus responses include pH response, temperature response, enzyme response, redox response, and so on (Thomas et al., 2020). The investigators synthesized a redox-responsive PEG-PUSeSe-PEG micelle that can exhibit excellent sensitivity to external redox stimuli (Ma et al., 2010). The micelles will dissociate because of their special response in the oxidation environment. The polymer can be used as a drug carrier for targeted drug release in an oxidation environment. Some scholars have developed a micellar platform for the co-delivery of an antiangiogenesis agent, axitinib (Axi), and a DNA intercalator, Dox (Zhang et al., 2020). This cross-linked micelle (DA-CM) could release Axi and Dox in tumor extracellular environment and intracellular lysosome compartments, respectively, in response to the dual pH stimulus. Compared with the traditional environmental response, light-sensitivity is a new type of intelligent response that uses light as an external stimulus to control the drug load and intelligent controlled release of polymer micelles. As a clean, non-invasive, external stimulus that does not alter internal conditions, light is less toxic and has fewer side effects (Zhao, 2007; Alvarez-Lorenzo et al., 2009; Beaute et al., 2019). By adjusting the wavelength and intensity, the time of action, location, and dose can be accurately controlled to achieve its controlled release of the drug (Chen et al., 2016; Hu et al., 2020; Yao et al., 2020).

**SCHEME 1:**
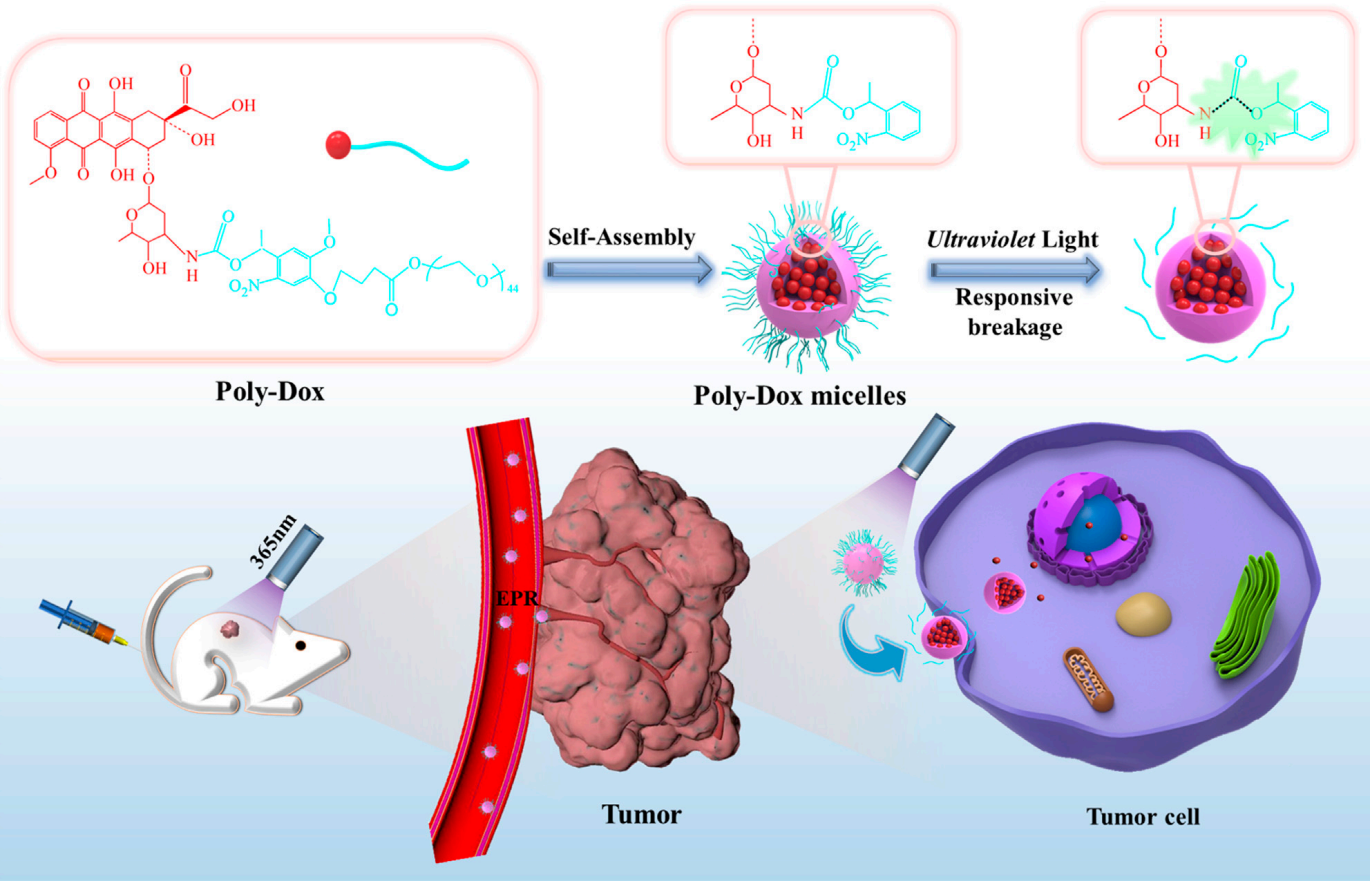
Schematic illustration of the self-assembly and responsive breakage of Poly-Dox micelles, and the process of tumor therapy.

Therefore, we have developed a light-responsive nano-micelle to achieve the efficient release of chemotherapeutic drugs in the tumor through ultraviolet irradiation. The light-responsive micelles with 27 nm diameter (Poly-Dox-M) are found to rapidly dissociate in several minutes upon UV light exposure and thus promote the effective release of the drug, followed by enhanced cellular uptake by tumor cells through endocytosis. Dox is connected with the inert structure PEG *via* UV-sensitive amide linkage to form a polymer, which then self-assembles into micelles in water (Hu et al., 2014). Due to the enhanced permeability and retention (EPR) effect of micellar polymers, Dox is selectively transferred to the tumor site (Chinen et al., 2015; Li et al., 2018; Riley et al., 2019). At the same time, the inert groups in the surface of nano-micelles can effectively reduce the drug uptake by other normal tissue cells, thereby effectively reducing the side effects of the drug. Then, UV irradiation on the tumor will break the amide bond on the micellar polymer structure, which will induce the quick release of Dox and enhance the cellular uptake by tumor cells, exerting its effective anti-cancer effect. In the present study, the successful synthesis of the polymer was confirmed by ^1^H-NMR. Several *in vitro* and *in vivo* experiments were performed. Meanwhile, the indexes indicated a great light-sensitive antitumor efficacy of micelle, which would be a highly promising therapeutic strategy for osteosarcoma (Scheme 1).

## Materials and Methods

### Materials

Polyethylene glycol (PEG, Mn = 2000), 4′-Hydroxy-3′-methoxyacetophenone, and doxorubicin hydrochloride (Dox) were purchased from Macklin Company, China. Dialysis bags (MWCO 3500) were purchased from Shanghai Green Bird Biological Co., Ltd. Ultraviolet laser (365 nm) was purchased from Changchun FMS Co., Ltd., China. All other reagents and solvents were purchased from Sinopharm Chemical Reagent Co., Ltd., China.

### Synthesis and Characterization of Poly-Dox

Using 4-hydroxy-3-methoxyacetophenone as raw material, the key intermediate 4-(4-acetyl-2-methoxy-5-nitro-phenoxy)-butyric acid 4 was obtained by the multi-step reaction, which was used as the module of the light response. Then the conjugated polymer Poly-Dox was obtained by a series of substitution reactions. The chemical structure was recorded by ^1^H NMR.

#### Preparation of Micelles

Firstly, conjugated polymer Poly-Dox was synthesized by a series of chemical reactions. A certain amount of Poly-Dox was dissolved in the DMSO solvent of 200 μl and oscillated in a water bath in an ultrasonic oscillator for 10 min. After it was completely dissolved, it was dropped under ultrasonic concussion and added to ultra-pure water (solution: ultra-pure = 1:7) to continue shaking for 10 min to obtain a clear and transparent solution. Put the prepared mixed solution into the dialysis membrane bag and seal it. Then the dialysis membrane bag was put into a magnetic agitator with ultra-pure water for 24 h to obtain Poly-Dox-M solution.

#### Characterization of Poly-Dox-M

The morphology was observed using TEM imaging (Hitachi H-600). The fluorescence spectra were measured using a multimode reader (BioTek Synergy). The fluorescence spectra changes of micelles were also evaluated after an ultraviolet light stimulus.

#### Ultraviolet Photo Response Characteristics of Poly-Dox-M

To evaluate the ultraviolet light sensitivity of Poly-Dox-M, the micelles were tested and compared with Free-Dox solution. Firstly, a micellar solution (1 mg/ml) was prepared, and the fluorescence spectra changes of the micellar solution after irradiation of 0, 3, 5, 7, 10, 13, 16, and 20 min were investigated by the multimode reader.

### Cell Culture

Osteosarcoma cell K7M2wt was purchased from the Chinese Academy of Science Cell Bank for Type Culture Collection (Shanghai, China). The cells were cultured in complete Dulbecco’s modified Eagle’s medium (DMEM) supplemented with 100 U/ml penicillin, 100 U/ml streptomycins, and 10% fetal bovine serum (FBS), and grown in a 37°C humidified environment containing 5% CO2. All experiments were conducted on cells in the logarithmic growth stage.

#### Determination of Cell Uptake

The uptake of Dox in Poly-Dox-M and Poly-Dox-M/UV solution by osteosarcoma cells was investigated by fluorescence microscope. Osteosarcoma cells growing in the logarithmic phase were collected and inoculated in 6-well plates with a density of 2 × 10^5^ cells per well, 3 ml per well. The culture medium was DMEM medium containing 10% fetal bovine serum and cultured overnight in a cell incubator at 37°C. The newly prepared Poly-Dox-M solution was added to the above 6-well plate and continued culture. The uptake time of 2, 4, and 6 h was set in 3 groups, and each group was done 3 times in parallel. The light group was irradiated with ultraviolet light at 5 min. At the corresponding time point, the cells were stained with the prepared staining solution for 10 min and then washed with PBS solution. Finally, the results were detected by fluorescence microscope.

#### *In Vitro* Cytotoxicity Assay

The cytotoxicity of Free-Dox, Poly-Dox-M, and Poly-Dox-M/UV was evaluated by the MTT assay (Zheng et al., 2015). K7M2wt cells were seeded in 96-well plates at a density of 5,000 cells per well in 100 μl DMEM containing 10% FBS and cultured for 24 h at 37°C. Then the cells were treated with a 100 μl culture medium containing a fixed amount of micelles for 48 h. After that, the medium was replaced with 100 μl of fresh DMEM and 10 μl MTT (5 mg/ml in PBS) and incubated for another 4 h. Then the medium was removed and 100 μl DMSO was added. The optical absorbance was measured at 570 nm of each well using an enzyme labeling instrument. To detect the light-responsive sensitivity, the cytotoxicity of Poly-Dox-M was analyzed after irradiated with ultraviolet light at 5 min. The cell viability (%) was determined by comparing the absorbance at 570 nm with control wells containing only the cell culture medium. All the cytotoxicity tests were conducted in triplicate.

#### Live-Dead Cell Staining

The cytotoxicity of PBS/UV, Free-Dox, Poly-Dox, and Poly-Dox/UV was examined by fluorescence microscope. K7M2wt cells growing in the logarithmic phase were collected and inoculated in 24-well plates with a density of 6 × 10^4^ cells per well, 1 ml per well. The culture medium was DMEM medium containing 10% fetal bovine serum and cultured overnight in a cell incubator at 37°C. The newly prepared solutions of PBS, Free-Dox, and Poly-Dox-M were diluted with medium and added to the above 24-well plate to continue the culture. the cells in the light group were irradiated with ultraviolet light for 5 min, and each group was done 3 times in parallel. At 24 h, the cells were stained with the prepared staining solution added to the 24-well plate for 10 min and then washed with PBS solution. Finally, the results were detected by fluorescence microscope.

### Animal Study

BALB/c nude mice (male, 19 ± 2g, 4–6 weeks) were purchased from Shanghai Slacker Company (Shanghai, China), and were randomly divided into 4 groups and housed in a controlled temperature with regular alternating cycles of light and darkness. All animal procedures were performed by national regulations and approved by the Animal Studies Ethical Committee of Jiaxing University School of Medicine, and the ethics number is JUMC 2019-004.

#### Establishment of Osteosarcoma Model in Nude Mice

The K7M2wt cells in the logarithmic phase were collected, mixed with serum-free medium, centrifuged with 1,500 r/min for 5 min, and the supernatant was discarded. Repeat this for 3 times to thoroughly remove the serum, and then add a certain amount of serum-free medium to obtain a cell suspension with a cell density of 4 × 10^8^ ml. Finally, 200 μl of cell suspension was subcutaneously injected into the axilla of nude mice, and about a month later, the subcutaneous tumor volume of nude mice was about 60 mm^3^, which could be used as a follow-up animal experimental model.

#### Immunohistochemistry

First of all, the prepared PBS, Free-Dox, and Poly-Dox-M solutions will be divided into four groups: PBS/UV, Free-Dox, Poly-Dox, and Poly-Dox/UV. Twelve tumor-bearing female nude mice were prepared, and the drugs of the above anti-tumor groups were injected into the tail vein according to the dose of Dox as 1.5 mg/kg 24 h later, the group that needed ultraviolet light was given light, and after 6 h of light, the nude mice were killed. Subsequently, six organs and tissues of the heart, liver, spleen, lung, kidney, and tumor of each nude mouse were removed and placed in pre-prepared formalin solution overnight. Subsequently, tissue sections were analyzed by hematoxylin and eosin (H&E), TUNEL staining, and Ki67 staining immunohistochemistry. Finally, the tissue sections of each organ were observed by an inverted microscope.

### Statistical Analysis

The data were presented as mean ± standard deviation. Analyses were performed with GraphPad Prism Software Version 8.00 (GraphPad Software, Inc., United States). The statistical differences between the two groups were analyzed according to the paired Student’s-test. Statistically, significances were presented when the *p*-value was less than 0.05 (*p* < 0.05).

## Results and Discussion

### Preparation and Characterization of Poly-Dox-M

**FIGURE 1:**
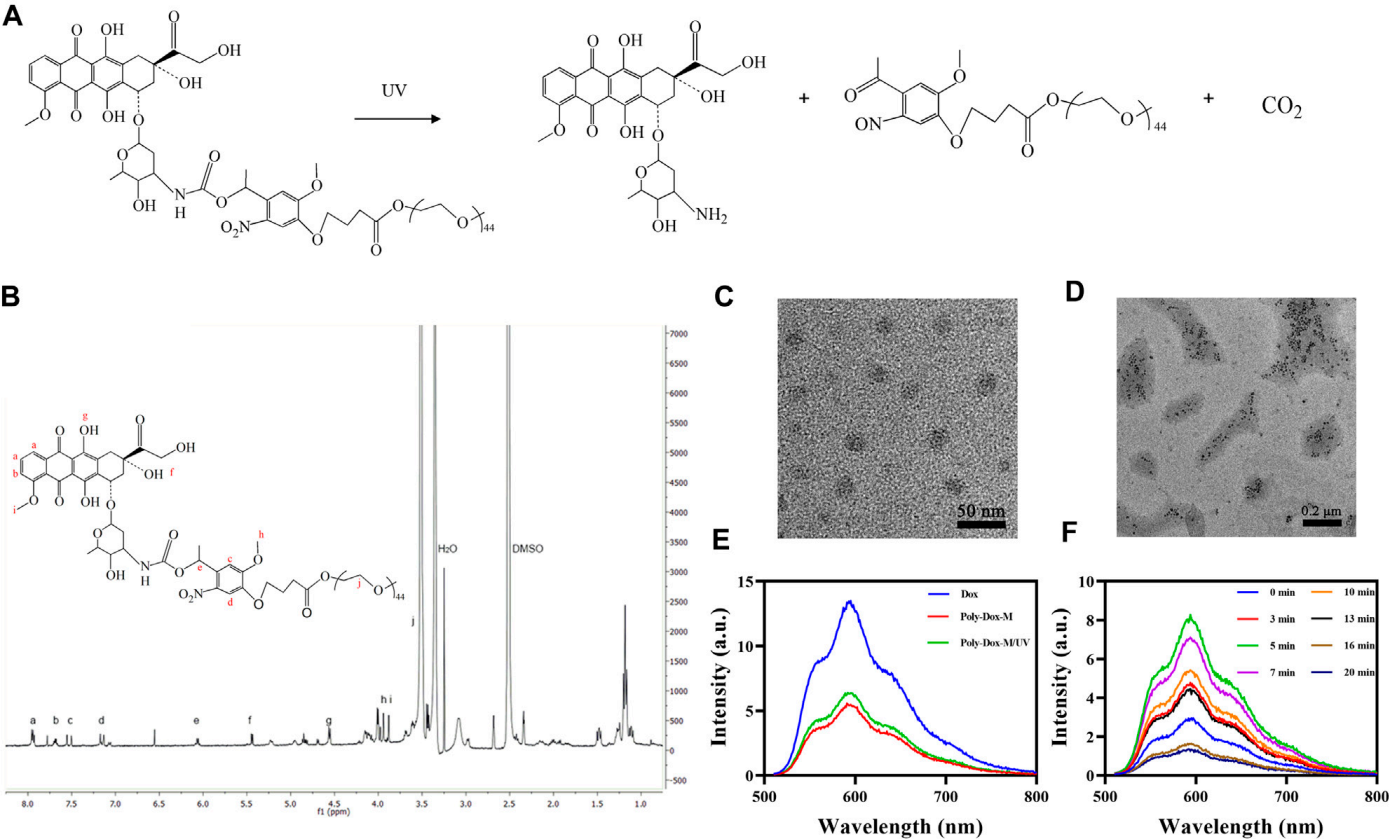
**(A)** Detailed depolymerization routes of Poly-Dox copolymer drug conjugates. **(B)** ^1^H-NMR spectra of Poly-Dox in DMSO-d6. **(C)** TEM image of Poly-Dox-M. **(D)** TEM image of disassembled Poly-Dox-M after UV irradiation. **(E)** Fluorescence intensity of Free-Dox, Poly-Dox-M, and Poly-Dox-M/UV. **(F)** The fluorescence intensity changes of Poly-Dox-M after UV irradiation of 0, 3, 5, 7, 10, 13, 16, and 20 min.

Briefly, we first synthesized the optical response module. Then the conjugated polymer Poly-Dox was obtained and characterized by a series of substitution reactions. As shown in Figure 1A, Under UV irradiation, the amide bonds in the polymer structure will break, resulting in the rapid release of the encapsulated drug. As illustrated in Figure 1B, we confirmed the successful synthesis of Poly-Dox with ^1^H-NMR. The Poly-Dox powdered polymers were dissolved in dimethyl sulfoxide (DMSO) and then self-assembled by progressive removal of the solvent through a dialysis solvent exchange process to obtain a Poly-Dox-M solution. The drug loading rate of the micelle was 13.62%. The size of micelles was characterized by TEM imaging (Figure 1C and Figure 1D). The results show that the micelles are spherical, uniform, and stable, and the average particle size is about 27 nm, which is more conducive to the effective enrichment of tumor tissue. More interestingly, the diameter of the micelles after UV irradiation is about 10 nm, which may enhance the penetration of the tumor. This variability happened indicating that UV light exposure effectively causes the responsive breakage of the polymer in the surface.

Subsequently, we prepared Poly-Dox-M and Free-Dox solutions and tested their spectra. As shown in Figure 1E, the spectra of the same concentration of Poly-Dox-M and Free-Dox solutions were dissimilar, which demonstrates that dox was successfully encapsulated in the micelles. The fluorescence intensity of Free-Dox at 601 nm wavelength was significantly higher than that of Poly-Dox-M, which indicating the quenching effect of the micelles (Hameed et al., 2018). Interestingly, the fluorescence intensity of Poly-Dox-M increased after ultraviolet irradiation of 3 min. It indicates that light effectively leads to structural changes in the nanoparticles which is related to the unique light-sensitivity of Poly-Dox-M. Reasonably, the micelles undergo cleavage upon UV irradiation, which causes the release of the encapsulated Dox and a consequent increase in the measured fluorescence intensity.

### Ultraviolet Light-Response Characteristics of Poly-Dox-M

To investigate the effect of different light times on Poly-Dox. We irradiated the prepared micellar solutions with 0, 3, 5, 7, 10, 13, 16, and 20 min of UV light and analyzed them with a multimode reader. As shown in Figure 1F, the fluorescence intensity of Poly-Dox-M increased gradually in the range of 0–5 min light duration and reached the peak at 5 min. However, after continuing to increase the light duration, the fluorescence intensity gradually decreased with the increase of time. This phenomenon was probably attributed to the quenching of the fluorescence of Dox after long-term UV irradiation (Pi et al., 2017). Notably, we can judge the time required for the complete cleavage of poly-dox by analyzing the changes of spectra. Thus, it can be seen that the best illumination duration of Poly-Dox prepared in this study should be controlled at about 5 min to exert its best killing effect.

### Cellular Uptake Study

**FIGURE 2:**
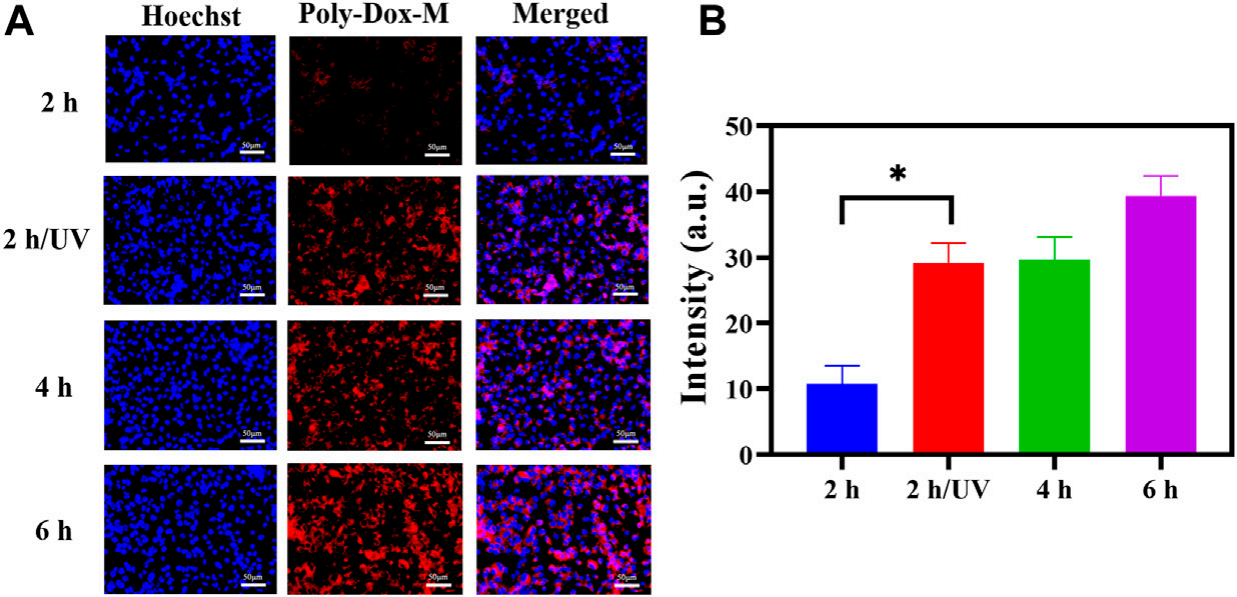
**(A)** K7M2-wt cells stained by Hoechst 33,342 after treated with Poly-Dox-M for 2, 4, and 6 h under UV irradiation or not. Blue signal reflects nucleus; red signal reflects Dox. Scale bar, 50 μm. **(B)** Their fluorescence intensity of Poly-Dox-M in different groups (Student’s *t*-test, *p < 0.05, **p < 0.01, ***p < 0.001).

In order to demonstrate the endocytosis of Poly-Dox-M, their cellular uptake was evaluated on K7M2wt cells. We first observed the fluorescent image of K7M2-wt cells treated with Poly-Dox-M for 2, 4, and 6 h incubation, and the results showed a time-dependent behavior of the cellular uptake. (Figures 2A,B). Then, in order to study the promotion of PEG shedding on the behavior of cellular uptake, we further observed the fluorescent image of K7M2-wt cells treated with Poly-Dox-M for 2 h incubation under 5 min UV irradiation. Notably, we observed a weak fluorescence intensity of Dox after 2 h incubation, but after another 5 min of UV irradiation, the fluorescence intensity of Dox was significantly enhanced. The results showed that the uptake ability of osteosarcoma cells to Dox was significantly enhanced after UV irradiation. This phenomenon arguably is caused by the PEG in the polymeric micelle structure shielding the adsorption of protein caps in the blood, thus weakening the uptake of cells. However, after light irradiation, the uptake of Dox by osteosarcoma cells was significantly enhanced. In one aspect, due to the cleavage of the amide bond in the micellar structure after illumination, the nanoparticles lose the shielding of the inert structure. On the other hand, osteosarcoma cells could take up the nanoparticles through efficient endocytosis, which further improved the uptake efficiency (Husseini and Pitt, 2008; Zhu et al., 2013; Chen et al., 2018).

### *In vitro* Cytotoxicity

**FIGURE 3:**
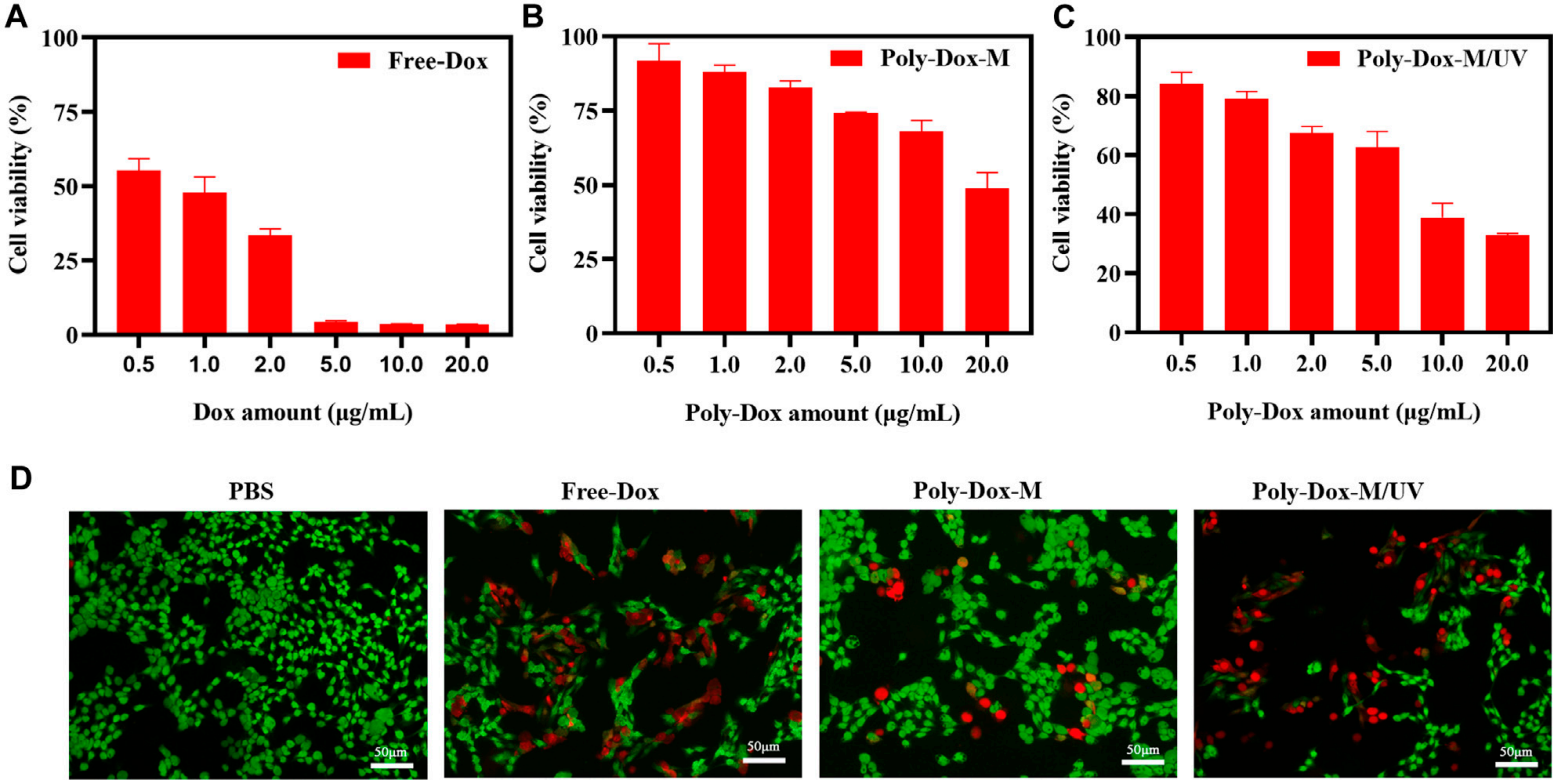
Cell viability of K7M2-wt cells treated with **(A)** Free-Dox. **(B)** Poly-Dox-M and **(C)** Poly-Dox-M under UV irradiation at a various dose of Dox or Poly-Dox. **(D)** K7M2-wt cells stained by calcein-AM (green) and PI (red) after 6 h incubation with PBS, free Dox, Poly-Dox-M under UV irradiation or not. Blue signal represents live cell, red signal represents dead cell. Scale bar: 50 μm.

To investigate whether the enhanced cellular uptake of the drug-loaded micelle could transform into increased anticancer activity, a cell cytotoxicity assay was performed. Firstly, the cytotoxicity of Free-Dox was evaluated (Figure 3A). All the samples showed concentration-dependent toxicity towards cells. Subsequently, to evaluate the cell damage of Poly-Dox-M, we incubated K7M2wt cells with Poly-Dox-M for 24 h, followed by 5 min light exposure (365 nm, 1.0 W cm^−2^) or not. In the presence of UV light irradiation, Poly-Dox-M exhibited IC_50_ values of approximately 14.1 μg ml^−1^ Dox (Figure 3B), principally resulting from chemotherapeutic cytotoxicity of Dox. Notably, Poly-Dox-M resulted in the cytotoxicity at IC_50_ levels of 6.1 μg ml^−1^ Dox under irradiation (Figure 3C). Obviously, Poly-Dox-M led to more dramatic cytotoxicity under irradiation, reasonably owing to their enhanced continuous drug release and cellular uptake in response after UV light.

### *In vivo* Anti-tumor Effect

**FIGURE 4:**
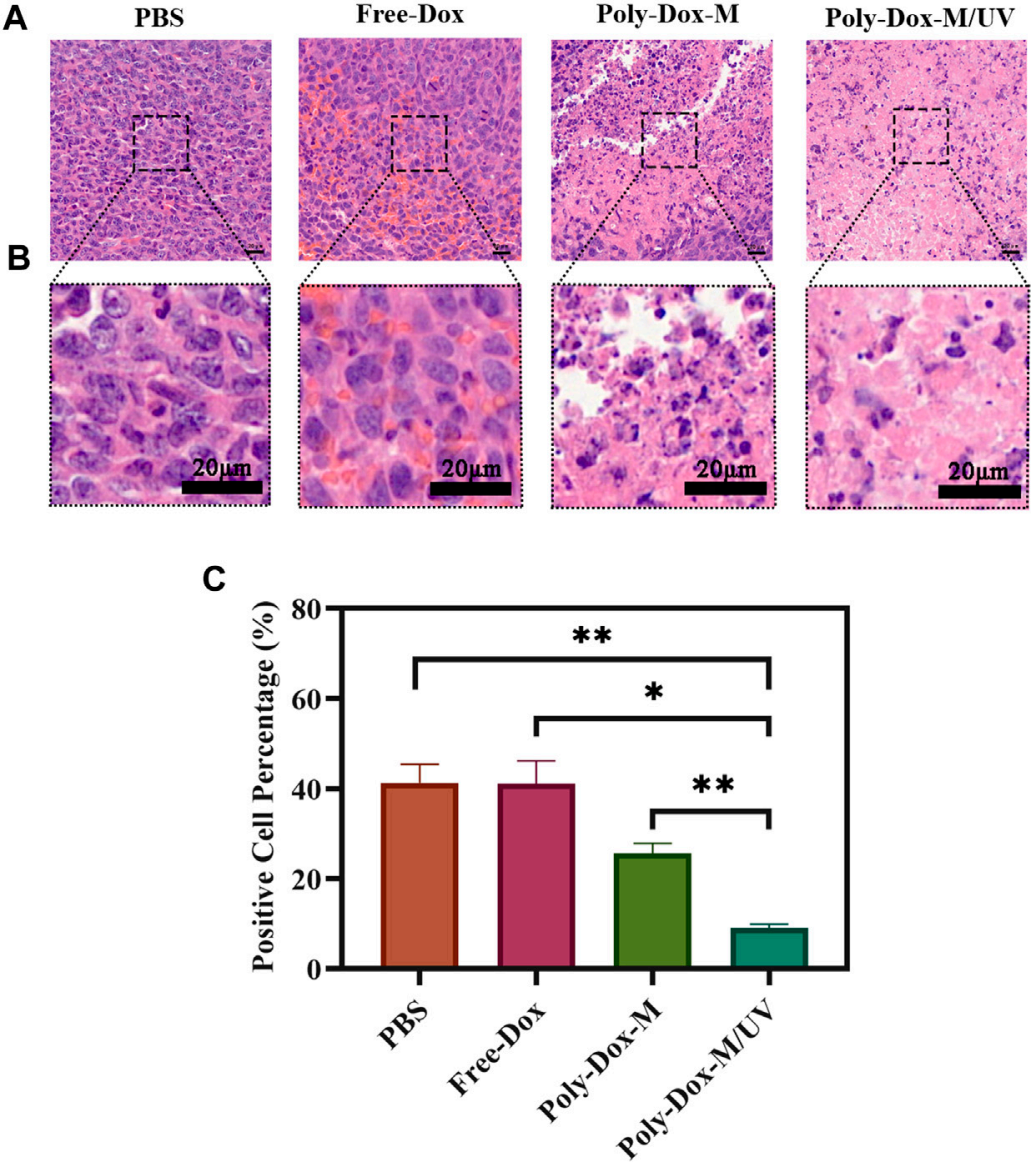
**(A)** H&E staining of tumor sections extracted from the mice treated with different samples. Scale bar: 20 μm. **(B)** An enlarged view of the boxed region below the corresponding image. Scale bar, 20 μm. **(C)** Image J analysis of H&E staining (Student’s *t*-test, *p < 0.05, **p < 0.01, ***p < 0.001).

**FIGURE 5:**
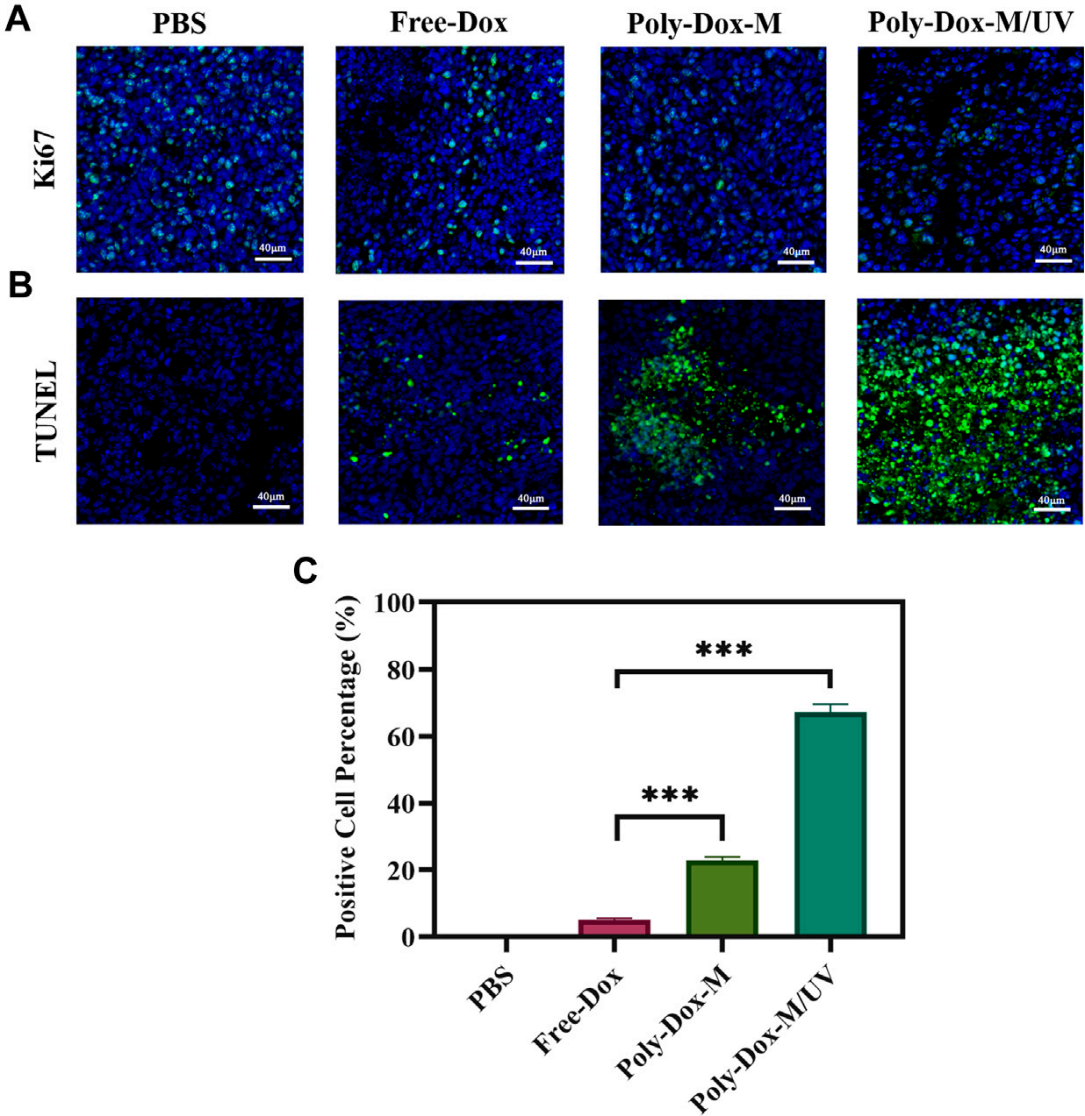
Immunofluorescent staining of tumor sections extracted from the mice treated with different samples. **(A)** Images of immunofluorescent staining for Ki67. Blue signals represent nucleus, green signals represent ki67 expression. **(B)** Images of Tunel staining. Blue signals represent nucleus, green signals represent apoptotic cells. **(C)** ImageJ analysis of TUNEL positive cell percentage. (Student’s *t*-test, *p < 0.05, **p < 0.01, ***p < 0.001).

**FIGURE 6:**
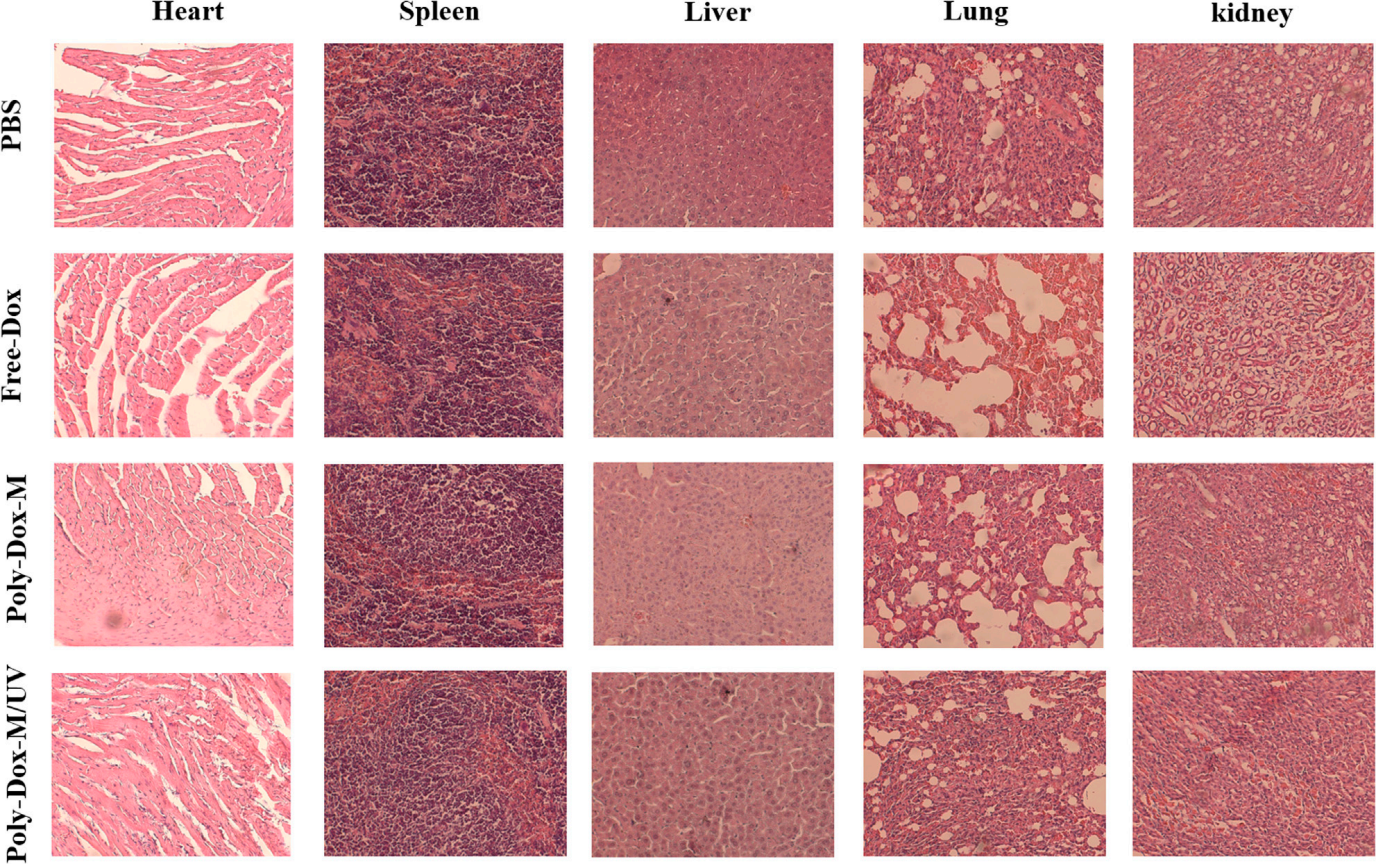
H&E staining of various tissues including heart, liver, spleen, lung and kidney extracted from the mice treated with PBS, Free-Dox, Poly-Dox-M under UV irradiation or not.

A xenograft model was constructed by implanting BALB/c nude mice with 4.0 × 10^8^ K7M2wt cells. To confirm the effect of Poly-Dox-M on killing K7M2wt xenografts *in vivo*, we establish Poly-Dox-M/UV group and different types of control groups, including Poly-Dox-M, Free-Dox, and PBS groups. H&E and Ki67 with immunofluorescence staining were performed to confirm the significantly enhanced antitumor activity and the best anti-tumor effect of the Poly-Dox-M system after UV light. Based on the image of HE staining (Figures 4A,B), we observed obvious cell atrophy and chromatin condensation. After the quantitative analysis by image J (Figure 4C), we further proved the anti-tumor effect of Poly-Dox-M under UV irradiation. Interestingly, compared with *in vitro* cytotoxicity, the enhancement in the *in vivo* anti-tumor activity by Poly-Dox-M under UV irradiation seems to be better, which may be attributed to the complex microenvironment of the tumor. As we all know, the tumor microenvironment is a complex system that contains various proteins, cells, etc. And it is reported that the cellular uptake of the nanoparticles is an energy-consuming process that requires cell membrane protein (Zhu et al., 2015). However, the composition of the culture medium in the *in vitro* cytotoxicity experiment is relatively simple, which lacks factors such as energy assistance from the external environment. In addition, it is reported that the tumor microenvironment is an acidic environment, and the release of doxorubicin is acidic responsive (Chen et al., 2014). Thus, the fast-release behavior of doxorubicin will quickly increase the concentration of doxorubicin in tumor, which may enhance their anti-tumor efficiency. Therefore, the effect *in vivo* will be better than that *in vitro*. As for the expression of ki67, the Poly-Dox-M/UV group had the fewest Ki67-positive nuclei (green stain) (Figure 5A). By contrast, the TUNEL assay showed the greatest increase in osteosarcoma cell apoptosis after Poly-Dox-M/UV group treatment (Figures 5B,C). The results demonstrate that Poly-Dox-M could effectively deliver Dox to tumors under the modulation of UV light and exert superior anti-tumor effects *in vivo*. Meanwhile, since H&E staining is the current common standard method for assessing the biosafety of the Nano drug-loaded particles applied in an *in vivo* experiment, we checked up those vital organs of a mouse injected with this nanoparticle, including the heart, spleen, liver, lung, and kidney, by H&E staining (Imai et al., 2015). Based on the histopathological analysis, there was no apparent evidence of tissue damage caused by this nanoparticle (Figure 6).

## Conclusion

In conclusion, we have successfully established a light-responsive nano-micelle drug delivery system (Poly-Dox-M) with ultrafast disassembly for highly effective chemotherapy. The PEG in the polymeric micelle structure can shield the adsorption of protein proteins in the blood circulation, maintaining a stable nanostructure for good tumor targeting and diminishing the systemic toxic effects of Dox. Also, drug-laden micelles can achieve effective enrichment at the tumor site through the EPR effect. Subsequently, after UV irradiation, the micelles at the tumor site underwent a responsive structural change, shedding the PEG and releasing the drug rapidly, making Dox more easily taken up by the tumor cells, thus further improving the efficiency of chemotherapy. The results of the study indicated that Poly-DOX-M possesses Sensitive light response characteristics, preferable cellular uptake, favorable bio-safety, and superior anti-cancer effect. The rational design of UV light-responsive nanoparticles represents a promising paradigm for highly efficient *in vivo* targeted delivery of anti-cancer agents and potentially acts as a complementary option to improving chemotherapy efficiency.

## Data Availability

The raw data supporting the conclusion of this article will be made available by the authors, without undue reservation.
